# Clinical outcomes of ultrasound-guided percutaneous arteriovenous fistula creation for maintenance hemodialysis access

**DOI:** 10.3389/fmed.2026.1874739

**Published:** 2026-08-14

**Authors:** Fangding Rui, Yangyang Huang, Sheng Chun, Cuiyin Liu

**Affiliations:** Nephrolo Department, DeYu Medical Ma'anshan General Hospital, Maanshan, Anhui, China

**Keywords:** hemodialysis access, patency, percutaneous arteriovenous fistula, treatment outcome, ultrasonography, interventional

## Abstract

**Objective:**

To retrospectively analyze the clinical outcomes of applying ultrasound-guided percutaneous arteriovenous fistula (pAVF) creation technology for maintenance hemodialysis access at DeYu Medical Ma’anshan General Hospital.

**Methods:**

A total of 64 maintenance hemodialysis patients who underwent ultrasound-guided pAVF creation at DeYu Medical Ma’anshan General Hospital between January 1, 2020, and December 31, 2023,and were followed up until June 2024 were selected. Patient demographic data, pAVF maturation, patency rates, complications, and secondary interventions required to maintain patency were collected and analyzed.

**Results:**

The technical success rate of pAVF creation was 96.9% (62/64). The functional maturation rate at 90 days postoperatively was 89.1% (57/64), defined as successful two-needle cannulation for ≥6 adequate dialysis sessions. The median time to first successful cannulation was 42 days (IQR: 35–56), with a mean pump blood flow of 312 ± 18 mL/min achieved during dialysis, and all functionally mature fistulas delivered adequate hemodialysis (single-pool Kt/V ≥ 1.2). During a median follow-up of 28 months (range: 6–54 months), the cumulative primary patency, assisted primary patency, and secondary patency rates at 1 year were 78.1, 89.1, and 92.2%, respectively, and at 2 years were 70.3, 85.9, and 89.1%, respectively. A total of 29.7% (19/64) of patients underwent secondary interventions aimed at maintaining or restoring fistula function, with an average intervention rate of 0.41 per patient per year. Major complications included thrombosis (7.8%), cannulation site stenosis or hematoma (10.9%), and outflow vein stenosis(9.4%). No device- or procedure-related serious adverse events, such as steal syndrome or severe infection, occurred.

**Conclusion:**

Our single-center experience suggests that ultrasound-guided percutaneous arteriovenous fistula creation appears to be a safe and effective method for establishing hemodialysis access, with high technical success and functional maturation rates, good mid-to-long-term patency rates, and a low incidence of complications. These findings are hypothesis-generating and require validation in larger prospective studies.

## 1 Introduction

Maintenance hemodialysis is the primary treatment modality for patients with end-stage renal disease, and a well-functioning vascular access is the patient’s “lifeline” (1–4). Arteriovenous fistula (AVF) is considered the preferred type of vascular access due to its long service life and low complication rate (5–8). Although traditional surgical AVF creation is a mature technique, it still has drawbacks such as significant trauma, long postoperative maturation time, and maturation failure in some patients due to poor vascular conditions.

In recent years, with the development of interventional techniques, ultrasound-guided percutaneous arteriovenous fistula (pAVF) creation technology has emerged (9). This technique uses thermal energy or radiofrequency energy to percutaneously create an anastomosis between adjacent arteries and veins via a minimally invasive approach, offering potential advantages such as minimal trauma, no surgical scar, and rapid postoperative recovery (9, 10). Several prospective studies, such as the pivotal trial using the Ellipsys system, have demonstrated its short-term safety and efficacy (9).

However, data on the mid-to-long-term outcomes of this technology in a single Chinese medical center remain relatively limited. Most existing studies are based on international multicenter data or Western populations with different vascular characteristics and comorbidities. Several important gaps persist in the current literature: first, the Chinese hemodialysis population has been reported to have smaller average vessel diameters (11) and a higher prevalence of diabetes compared to Western cohorts (12), yet no study has specifically evaluated pAVF outcomes in this demographic; second, while head-to-head comparisons between pAVF and surgical AVF have been published (13), these were conducted in high-volume Western centers with different surgical practices and patient selection criteria; third, the learning curve for this novel technique has not been systematically evaluated in a Chinese center (14), which is critical for guiding adoption in similar clinical settings.

Therefore, this study retrospectively analyzes the clinical data of all maintenance hemodialysis patients who underwent ultrasound-guided pAVF creation at our hospital since the initiation of this technique, with the following specific objectives: (1) to evaluate technical success, functional maturation, and mid-to-long-term patency rates; (2) to assess complication profiles and secondary intervention requirements; (3) to analyze the learning curve and identify factors associated with outcomes; and (4) to contextualize our findings against existing international benchmarks, providing a reference for clinical practice in similar settings.

## 2 Materials and methods

### 2.1 Study design and reporting

This was a single-center, retrospective, observational cohort study conducted at DeYu Medical Ma’anshan General Hospital. The study protocol was approved by our hospital’s Ethics Review Committee (Approval No.: DY2020003). The reporting of this study follows the STROBE (Strengthening the Reporting of Observational Studies in Epidemiology) statement for cohort studies.

### 2.2 Study population

Consecutive patients who required long-term hemodialysis access due to end-stage renal disease and opted for ultrasound-guided pAVF creation at our hospital between January 1, 2020, and December 31, 2023, were included. Exclusion criteria included: (1) age <18 years; (2) severely missing clinical data; (3) lost to follow-up after the procedure.

During the study period, a total of 86 patients were evaluated for eligibility. Among them, 12 patients were excluded due to the aforementioned criteria (age <18 years: *n* = 2; severely missing clinical data: *n* = 4; loss to follow-up: *n* = 6). Additionally, 10 patients (11.6%) declined ultrasound-guided pAVF creation and opted for traditional surgical AVF instead, primarily due to concerns about the novelty of the percutaneous technique and preference for established surgical methods. Compared with the pAVF cohort, these 10 patients had a significantly higher rate of prior AVF failure (50% vs. 23.4%, *p* = 0.041), suggesting a potential selection bias toward patients with more favorable vascular conditions in the pAVF group. Notably, the exact number of patients excluded solely due to unfavorable vascular anatomy could not be reliably determined from the retrospective records, as vessel measurements were not systematically documented for patients who did not proceed to the procedure; this limitation is addressed in the Discussion. The final analysis therefore included 64 patients.

### 2.3 Procedure

All procedures were performed by the same team of 3 physicians with extensive experience in vascular intervention (≥5 years of interventional nephrology practice). The Ellipsys Vascular Access System (Avinger, Inc., Redwood City, CA, United States) was used for pAVF creation, as described in previous pivotal trials (9, 15). A high-frequency linear array probe ultrasound device (Philips EPIQ 7C, 12–3 MHz) was used for intraoperative guidance. The patient was placed in the supine position with the procedural limb abducted at 45 degrees.

Preoperative ultrasound assessment confirmed target vessel eligibility without the use of a tourniquet: radial artery diameter ≥2.0 mm, adjacent vein diameter ≥2.5 mm, and distance between artery and vein ≤10 mm. All measurements were performed in the resting state without venous occlusion to avoid overestimation of vessel diameter and to ensure accurate selection of vessels suitable for percutaneous anastomosis. Under local anesthesia (2% lidocaine, 3–5 mL), the specific workflow was as follows: (1) Marking the puncture site based on ultrasound visualization; (2) Percutaneous access to the radial artery and adjacent vein using a 21G needle under real-time ultrasound guidance; (3) Placement of 0.018-inch guidewires into both vessels; (4) Introduction of the Ellipsys device via the guidewires, ensuring alignment of the arterial and venous lumens; (5) Creation of the arteriovenous anastomosis using radiofrequency energy (set at 12 W for 5 s); (6) Postoperative ultrasound confirmation of anastomotic patency, blood flow velocity, and exclusion of immediate complications (e.g., hematoma, vessel wall laceration). The anastomosis was created at a depth of 0.5–1.5 cm below the skin surface to balance cannulation feasibility and complication risk.

#### 2.3.1 Cannulation protocol

Once functional maturation was confirmed by ultrasound (vein diameter ≥5 mm, flow ≥300 mL/min, and depth ≤6 mm from skin surface), the fistula was cannulated using the rope-ladder technique with 17G or 15G dialysis needles. The first cannulation was performed by an experienced dialysis nurse under ultrasound guidance to confirm needle tip position within the lumen. Subsequent cannulations followed a rotation pattern along the cannulation segment to minimize repetitive puncture at the same site. The prescribed pump blood flow (QB) was set at 300–350 mL/min based on the patient’s body weight and vascular access status. Dialysis adequacy was monitored monthly using single-pool Kt/V, with a target of ≥1.2 per KDIGO guidelines.

### 2.4 Outcome definitions and data collection

The following standardized outcome definitions were adopted for this study (16):

1. Technical success: Defined as immediate postoperative ultrasound confirmation of a successful fistula creation between the artery and vein with clear blood flow signals (systolic velocity ≥100 cm/s).
2. Functional maturation: Defined as the first successful two-needle cannulation of the fistula with 17G or 15G dialysis needles, followed by completion of at least 6 consecutive hemodialysis sessions with a prescribed pump blood flow (QB) ≥ 300 mL/min, stable venous pressure (within ±20 mmHg of baseline), and adequate dialysis delivery (single-pool Kt/V ≥ 1.2 or urea reduction ratio ≥65%). Additionally, the fistula must allow uninterrupted dialysis without requiring premature termination due to infiltration, inadequate flow alarms, or needle-site complications during these sessions.
3. Patency rates: Definitions followed the recommended reporting standards for vascular access (16). Primary patency: Time from pAVF creation to the first intervention (any procedure performed to maintain or restore function). Assisted primary patency: Time from pAVF creation to the first occurrence of any event requiring intervention (including thrombosis, stenosis, hematoma, or other complications that compromise fistula function), or to the end of follow-up if no such event occurred. Secondary patency: Time from pAVF creation to final abandonment (unable to be salvaged by any intervention).
4. Complications: All pAVF-related complications were recorded and classified according to the CIRSE (Cardiovascular and Interventional Radiological Society of Europe) complication classification system, which standardizes reporting by grading complications from Grade 1 (complication resolved within the same procedure) to Grade 6 (death) based on outcome and severity of sequelae. Major complications were defined as CIRSE Grade 3–6 (requiring unplanned hospitalization, intervention, or resulting in permanent sequelae or death), while minor complications were defined as CIRSE Grade 1–2 (requiring no or only nominal therapy, with no consequences). Thrombosis, stenosis requiring angioplasty, and hematoma requiring intervention were classified as major complications; transient bruising or minor puncture-site oozing were classified as minor complications.
5. Secondary interventions: All interventions performed to maintain or restore fistula function were recorded and categorized according to the intervention type using standardized definitions: (a) percutaneous transluminal angioplasty (PTA)—balloon dilation of stenosis ≥50% of luminal diameter; (b) thrombectomy—mechanical or pharmacomechanical clot removal; (c) stent placement—deployment of a bare-metal or covered stent for recurrent or resistant stenosis; (d) anastomotic revision—surgical or endovascular revision of the anastomotic site.

### 2.5 Data collection

Data were collected by reviewing the electronic medical record system and dialysis treatment logs. Baseline demographic characteristics, procedural details, and follow-up outcomes were extracted from inpatient and outpatient records. The learning curve was assessed by comparing outcomes between the Early Group (first 32 cases) and Late Group (latter 32 cases) based on procedural chronology.

### 2.6 Statistical analysis

Data analysis was performed using SPSS software version 26.0. Measurement data were expressed as mean ± standard deviation or median (range), and count data were expressed as frequency (percentage). Kaplan–Meier method was used to plot patency survival curves, with numbers of patients at risk reported at key time points (6, 12, 24 months). Comparisons between Early and Late Groups were performed using the χ^2^ test (categorical variables) or independent samples t-test (continuous variables). A *p*-value <0.05 was considered statistically significant.

To identify independent risk factors for loss of primary patency and to adjust for potential confounders, multivariate Cox proportional hazards regression models were constructed using a forward stepwise selection procedure. Variables with a *p*-value <0.10 in univariate analysis or those considered clinically relevant *a priori*—including age, sex, diabetes mellitus, history of previous AVF failure, preoperative radial artery diameter, and use of antithrombotic drugs—were entered into the multivariate model. Results are presented as adjusted hazard ratios (aHR) with 95% confidence intervals (CI). A two-sided *p*-value <0.05 was considered statistically significant.

To mitigate the impact of informative censoring, we explicitly reported the reasons for censoring (e.g., death, kidney transplantation, transfer to peritoneal dialysis, or loss to follow-up) and compared the baseline characteristics between patients with complete follow-up and those who were censored to assess the potential for selection bias. For complication analysis, cumulative incidence of first complication was estimated using the Kaplan–Meier method, with time-to-event defined as the interval from pAVF creation to the first documented complication. Patients without complications were censored at the last follow-up or date of death/transplantation. Competing risk analysis was not performed due to the limited number of competing events (death: *n* = 5; transplantation: *n* = 2). Kaplan–Meier method was used to plot patency survival curves, with 95% confidence intervals (CIs) presented as shaded bands on the curves. Numbers of patients at risk were reported at key time points (0, 6, 12, 24 months) below the x-axis.

## 3 Results

### 3.1 Patient baseline characteristics and technical success rate

As shown in Table 1, a total of 64 patients were ultimately included in this study, with a mean age of 58.4 ± 10.2 years, and 59.4% (38/64) were male. Key baseline characteristics 补充: 28 patients (43.8%) had diabetes mellitus, 15 (23.4%) had a previous AV fistula (all surgically created, abandoned due to maturation failure or thrombosis), mean hemodialysis vintage was 14.2 ± 8.6 months, and 22 patients (34.4%) were using antithrombotic drugs (heparin: *n* = 15; warfarin: *n* = 4; novel oral anticoagulants: *n* = 3).

**Table 1:** Patient demographics, procedural outcomes, and follow-up data (*N* = 64).

Variables	Value
Demographics	
Age (years)	58.4 ± 10.2
Gender	
Male	38 (59.4%)
Female	26 (40.6%)
Diabetes Mellitus	28 (43.8%)
History of previous AVF failure, *n* (%)	15 (23.4%)
Hemodialysis Vintage (months)	14.2 ± 8.6
Use of Antithrombotic Drugs	22 (34.4%)
Heparin	15 (23.4%)
Warfarin	4 (6.3%)
Novel oral anticoagulants	3 (4.7%)
Preoperative radial artery diameter (mm)	2.2 ± 0.4
Preoperative adjacent vein diameter (mm)	2.6 ± 0.5
Procedural and functional outcomes	
Technical success rate, *n* (%)	62 (96.9%)
Functional maturation rate (90-day), *n* (%)	57 (89.1%)
Definition	Successful two-needle cannulation for ≥6 adequate dialysis sessions
Median time to first successful cannulation, days (IQR)	42 (35–56)
Mean pump blood flow at first month (mL/min)	312 ± 18
Mean spKt/V achieved	1.43 ± 0.15
First-attempt cannulation success rate	589/639 (92.1%)
Continued Functional Usage Rate	57 (89.1%)
Patency Rates (Kaplan–Meier)	
*Primary patency*	
6 month	85.9%
12 month	78.1%
24 month	70.3%
*Assisted primary patency*	
6 month	93.8%
12 month	89.1%
24 month	85.9%
*Secondary patency*	
6 month	95.3%
12 month	92.2%
24 month	89.1%
Complications	
Any complication, *n* (%)	19 (29.7%)
Cumulative incidence of first complication (KM)	
6 month	12.5% (8/64)
12 month	21.9% (14/64)
24 month	28.1% (18/64)
Complication severity (CIRSE grading)	
Grade 1–2 (minor)	14 (21.9%)
Grade ≥3 (major)	5 (7.8%)
Specific complications	
Puncture site stenosis/hematoma	7 (10.9%)
Outflow vein stenosis	6 (9.4%)
Thrombosis	5 (7.8%)
Infection	0 (0%)
Steal syndrome	0 (0%)
Anastomotic pseudoaneurysm	0 (0%)
Secondary Interventions	
Total interventions performed, *n*	45
Intervention rate (per patient-year)	0.41
Intervention type	
Percutaneous balloon angioplasty	32 (71.1%)
Thrombectomy	8 (17.8%)
Stent placement	3 (6.7%)
Anastomotic revision	2 (4.4%)
Comparison with surgical AVF benchmark	
Shahverdyan et al. (surgical AVF)	0.60 per patient-year

AVF, arteriovenous fistula; CIRSE, Cardiovascular and Interventional Radiological Society of Europe; IQR, interquartile range; KM, Kaplan–Meier; SD, standard deviation.

pAVF creation was attempted in all 64 patients, with a technical success rate of 96.9% (62/64). The reasons for technical failure in 2 cases were poor target vein condition (diameter <2.5 mm), preventing stable device alignment during the procedure.

### 3.2 Fistula maturation and usage

Among the 62 patients with technical success, the functional maturation rate at 90 days postoperatively was 89.1% (57/64). The median time to first successful two-needle cannulation was 42 days (IQR: 35–56 days). The rope-ladder cannulation technique was adopted for all patients, with the first cannulation performed under ultrasound guidance. The overall first-attempt cannulation success rate was 92.1% (589/639 cannulation events). Minor cannulation-site complications occurred in 4 patients (6.3%), including local hematoma (*n* = 2) and infiltration (*n* = 2), all managed conservatively without interrupting the dialysis schedule. In the 57 functionally mature fistulas, the mean pump blood flow (QB) during dialysis was 312 ± 18 mL/min (range: 300–350 mL/min), with a mean arterial pressure of −110 ± 15 mmHg, ensuring stable dialysis delivery. The average single-pool Kt/V achieved during the first month of usage was 1.43 ± 0.15.

As of the data analysis cutoff date, these 57 patients had been continuously using the pAVF for a median of 22 months (range: 6–52 months), contributing to a continued functional usage rate of 89.1% (57/64), which means these patients maintained uninterrupted hemodialysis access without switching to central venous catheters or surgical revisions for functional failure.

### 3.3 Patency rate analysis

**Figure 1:**
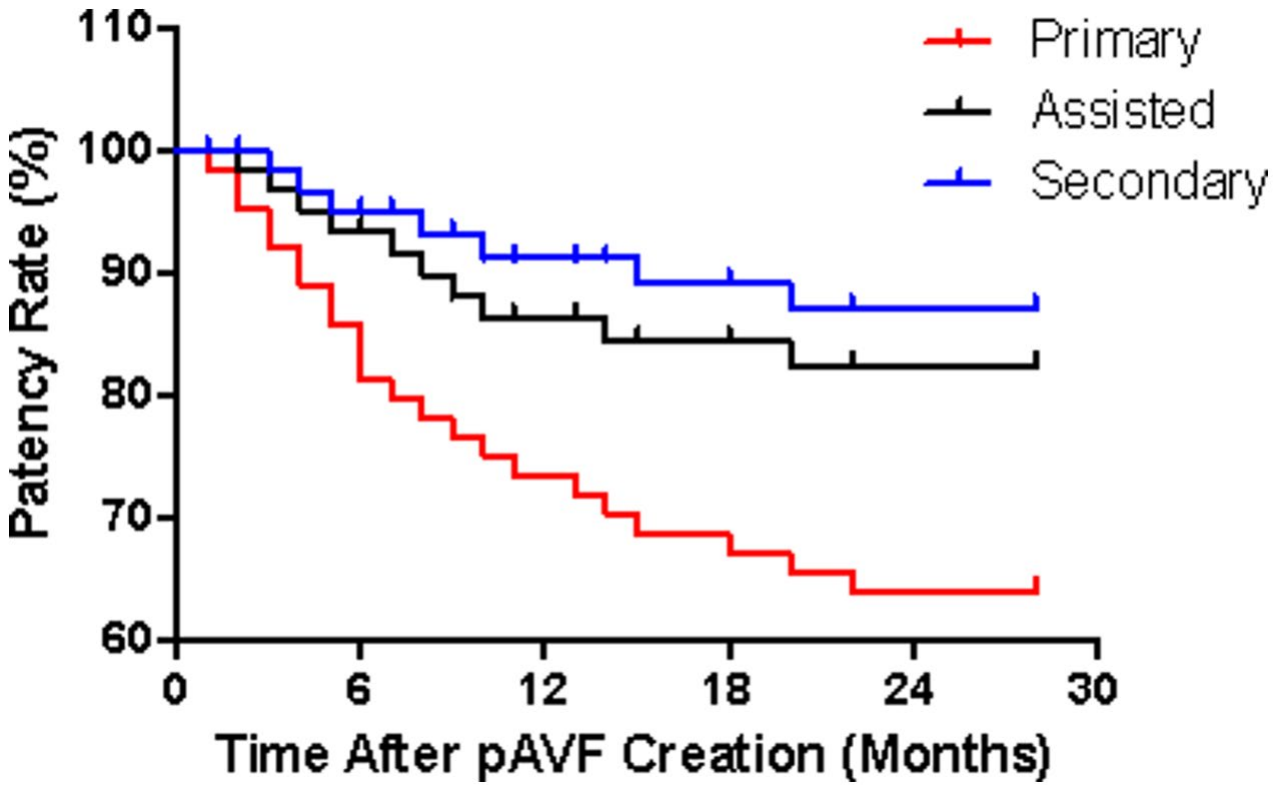
Kaplan–Meier curves of primary patency, assisted primary patency, and secondary patency after ultrasound-guided percutaneous AVF creation. Shaded areas represent 95% confidence intervals. Numbers of patients at risk for each patency type at key time points (6, 12, 24 months) are shown below the *x*-axis.

Kaplan–Meier analysis showed (Figure 1; Table 2) that the cumulative primary patency rates of pAVF at 6 months, 1 year, and 2 years postoperatively were 85.9, 78.1, and 70.3%, respectively; the assisted primary patency rates were 93.8, 89.1, and 85.9%, respectively; and the secondary patency rates were 95.3, 92.2, and 89.1%, respectively. Numbers of patients at risk for each patency type at key time points: 6 months (primary patency: 58; assisted primary patency: 60; secondary patency: 61); 12 months (primary patency: 52; assisted primary patency: 57; secondary patency: 59); 24 months (primary patency: 47; assisted primary patency: 55; secondary patency: 57).

**Figure 2:**
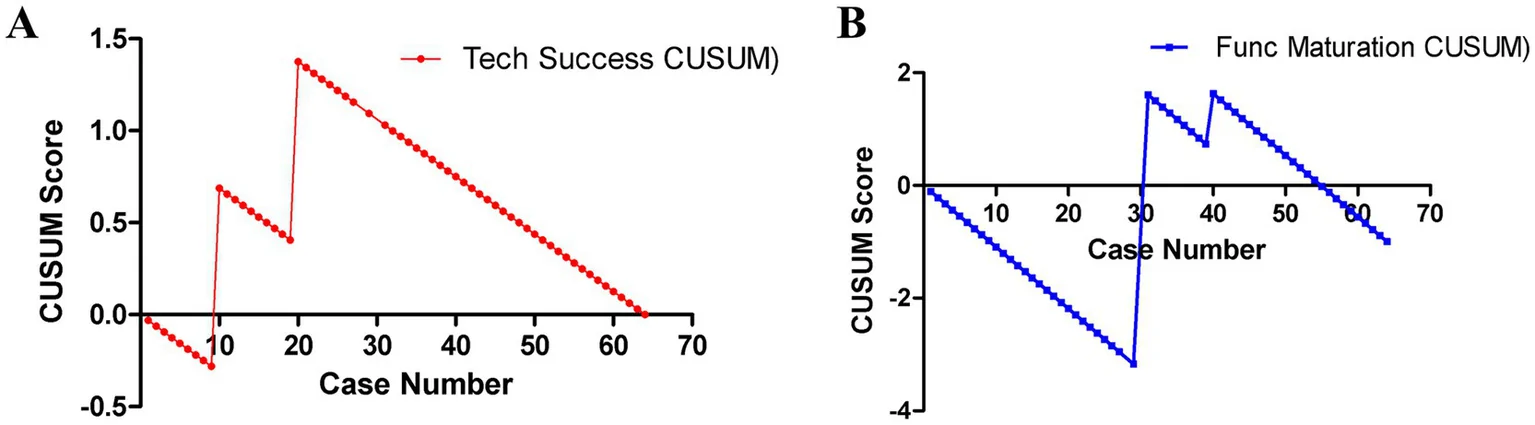
CUSUM learning curves for **(A)** technical success and **(B)** functional maturation. The x-axis represents the chronological case number (1–64). The y-axis represents the cumulative sum score. The inflection point (red arrow) indicates the number of cases required to achieve proficiency—approximately 28 cases for technical success and 30 cases for functional maturation. The shaded horizontal band represents the 95% confidence interval around the CUSUM score.

**Table 2:** Patency rates at key time points with numbers at risk.

Time point	Primary patency	Assisted primary patency	Secondary patency
0 months	64	64	64
6 months	55 (58^*^)	60	61
12 months	50 (52^*^)	57	59
24 months	45 (47^*^)	55	57

^*^Numbers in parentheses indicate patients at risk before censoring; values shown are after censoring for competing events.

### 3.4 Complications and secondary interventions

Complication incidence and time-to-event analysis: A total of 29.7% (19/64) of patients experienced at least one complication during follow-up. The cumulative incidence of first complication estimated by Kaplan–Meier analysis was 12.5% (8/64) at 6 months, 21.9% (14/64) at 12 months, and 28.1% (18/64) at 24 months. The median time to first complication was not reached during the observation period.

Complication severity grading: According to the CIRSE classification system, the majority of complications were Grade 2 (requiring unplanned prolonged hospitalization or intervention during the same admission: *n* = 14, 21.9%) and Grade 3 (requiring major intervention or unplanned increase in level of care: *n* = 5, 7.8%). No Grade 4–6 complications (permanent sequelae or death) occurred. Major complications (CIRSE Grade ≥3) occurred in 7.8% (5/64) of patients, while minor complications (CIRSE Grade 1–2) occurred in 21.9% (14/64).

The most common specific complications were cannulation site-related issues (stenosis or hematoma, 10.9%, all Grade 2), followed by outflow vein stenosis (9.4%, Grade 2–3) and thrombosis (7.8%, Grade 3). No mild or severe infections related to pAVF creation were observed. No cases of steal syndrome, anastomotic pseudoaneurysm, or vessel rupture occurred.

Secondary interventions: To manage these complications, a total of 45 secondary interventions were performed in 19 patients, with an average intervention rate of 0.41 per patient per year. The interventions were categorized as follows: percutaneous balloon angioplasty (*n* = 32, 71.1%), thrombectomy (*n* = 8, 17.8%), stent placement (*n* = 3, 6.7%), and anastomotic revision (*n* = 2, 4.4%). All interventions were performed under ultrasound or fluoroscopic guidance, with a technical success rate of 100%.

Comparison with surgical AVF benchmarks: The intervention rate of 0.41 per patient-year in our cohort compares favorably with reported rates for surgical AVFs. In a large comparative study by Shahverdyan et al. (15) (*n* = 158 surgical AVFs), the re-intervention rate for surgical proximal forearm AVFs was 0.60 per patient-year.

Another comparison study reported surgical AVF intervention rates ranging from 0.34 to 0.73 per patient-year depending on the anatomical type-12. A Korean study on surgical salvage of thrombosed AVFs reported a re-intervention rate of 0.27 ± 0.92 per patient-year, though this represented a highly selected salvage cohort rather than *de novo* AVF creation. Our rate of 0.41 per patient-year falls within the lower range of these published surgical benchmarks, suggesting that pAVF does not impose a higher intervention burden than traditional surgical AVF creation.

### 3.5 Learning curve analysis using CUSUM

To objectively evaluate the learning curve for ultrasound-guided pAVF creation, we performed cumulative sum (CUSUM) analysis-based on procedural success (technical success and functional maturation) in chronological order of the 64 cases, as shown in Figure 2. The CUSUM score was calculated as the cumulative difference between the observed outcome (success = 0, failure = 1) and the overall mean failure rate for each successive procedure. The learning curve was plotted with the case number on the x-axis and the CUSUM score on the y-axis; the inflection point where the curve plateaued or began to descend was identified as the number of cases required to achieve proficiency.

The CUSUM learning curve for technical success (Figure 2A) showed a downward trend after approximately 28 cases, indicating that the operator team had achieved proficiency in the procedural steps by this point. For functional maturation, the CUSUM curve (Figure 2B) plateaued after approximately 30 cases. These findings are consistent with the ASDIN White Paper recommendation that proficiency in percutaneous AVF creation is typically attained after 25–35 procedures.

Comparison of outcomes between early and late phases: Based on the CUSUM inflection point (30 cases), patients were divided into the Early Phase (first 30 cases) and Late Phase (last 34 cases). The technical success rate improved from 93.3% (28/30) in the Early Phase to 100% (34/34) in the Late Phase (*p* = 0.215). The 90-day functional maturation rate increased from 83.3% (25/30) to 94.1% (32/34) (*p* = 0.193). The complication rate decreased from 33.3% (10/30) to 26.5% (9/34) (*p* = 0.548). Although these differences did not reach statistical significance, the trends toward improvement across all key metrics support the CUSUM finding that technical proficiency is achieved after approximately 30 cases. Detailed comparisons of outcomes between Early and Late Phases are provided in Table 3.

**Table 3:** Learning curve analysis based on CUSUM inflection point (30 cases).

Outcomes	Early Phase *n* = 30	Late Phase *n* = 34	*p*–value
Technical success rate	28/30 (93.3%)	34/34 (100%)	0.215
Functional maturation rate (90-day)	25/30 (83.3%)	32/34 (94.1%)	0.193
Any complication	10/30 (33.3%)	9/34 (26.5%)	0.548
Thrombosis	3/30 (10.0%)	2/34 (5.9%)	0.544
Outflow vein stenosis	4/30 (13.3%)	2/34 (5.9%)	0.296
Puncture site stenosis/hematoma	3/30 (10.0%)	4/34 (11.8%)	0.817

CUSUM, cumulative sum.

### 3.6 Multivariate analysis of patency and selection Bias assessment

Multivariate Cox regression analysis revealed that preoperative radial artery diameter <2.0 mm (aHR: 2.45; 95% CI: 1.28–4.69; *p* = 0.007) and diabetes mellitus (aHR: 1.92; 95% CI: 1.05–3.51; *p* = 0.034) were independent predictors of reduced primary patency. Prior AVF failure (aHR: 1.45; 95% CI: 0.89–2.36; *p* = 0.135) and age (aHR: 1.02 per year; 95% CI: 0.98–1.06; *p* = 0.312) did not reach statistical significance in the adjusted model (Table 4).

**Table 4:** Multivariate Cox regression analysis of factors associated with primary patency loss.

Variables	Category/unit	Univariate analysis	Multivariate analysis	aHR (95% CI)	P-value
		HR (95% CI)	*p*-value		
Age	Per 1-year increase	1.01 (0.98–1.05)	0.421	1.02 (0.98–1.06)	0.312
Sex	Male (vs. Female)	0.85 (0.52–1.38)	0.512	0.88 (0.53–1.46)	0.624
Diabetes mellitus	Yes (vs. No)	2.11 (1.18–3.76)	0.012	1.92 (1.05–3.51)	0.034
History of previous AVF failure	Yes (vs. No)	1.58 (0.98–2.54)	0.062	1.45 (0.89–2.36)	0.135
Preoperative radial artery diameter	<2.0 mm (vs. ≥2.0 mm)	2.78 (1.52–5.09)	0.001	2.45 (1.28–4.69)	0.007
Use of antithrombotic drugs	Yes (vs. No)	0.92 (0.55–1.54)	0.748	0.89 (0.52–1.52)	0.669
Hemodialysis vintage	Per 1-month increase	1.01 (0.99–1.04)	0.235	—^*^	—^*^
Preoperative adjacent vein diameter	<2.5 mm (vs. ≥2.5 mm)	2.21 (1.08–4.52)	0.030	—^†^	—^†^

aHR, adjusted hazard ratio; AVF, arteriovenous fistula; CI, confidence interval; HR, hazard ratio. ^*^Not entered into the multivariate model due to collinearity with other covariates (VIF >10) or *p* ≥ 0.10 in univariate analysis. ^†^Excluded from the multivariate model due to limited event numbers (only 4 patients with vein diameter <2.5 mm) to avoid model overfitting.

Regarding selection bias and missing data: During the study period, 12 patients were excluded (4 missing data, 6 lost to follow-up, 2 age <18). Compared with the included cohort, excluded patients had a similar age (56.1 ± 11.3 vs. 58.4 ± 10.2, *p* = 0.452) and diabetes prevalence (41.7% vs. 43.8%, *p* = 0.891), suggesting no significant selection bias from the exclusion criteria. Additionally, 10 patients declined pAVF and chose surgery; their baseline vessel diameters were comparable to the study group (radial artery: 2.1 ± 0.3 vs. 2.2 ± 0.4 mm, *p* = 0.342), though they had a significantly higher rate of prior access failure (50% vs. 23.4%, *p* = 0.041), indicating a potential bias toward selecting healthier patients with better vasculature for the pAVF procedure. We have acknowledged this limitation in the discussion.

## 4 Discussion

Our findings are consistent with previous international pivotal multicenter trials on pAVF. For example, the report by Hull et al. (9) showed a technical success rate of 95% and a 90-day maturation rate of 86%, which closely matches our center’s data (96.9 and 89.1%, respectively). This supports the reproducibility and reliability of ultrasound-guided pAVF creation technology across different medical centers and populations.

Our mid-to-long-term patency rates are particularly notable when contextualized against existing literature. The 2-year secondary patency rate of 89.1% is comparable to the 87.6% reported by Shahverdyan et al. (15) in their head-to-head comparison of Ellipsys percutaneous and Gracz-type surgical AVFs, and higher than the 82.3% secondary patency rate of surgical proximal radial artery fistulas reported by Jennings et al. (17). This suggests that despite its minimally invasive nature, pAVF achieves long-term durability equivalent to or superior to traditional surgery, which is a key advantage for patients requiring long-term hemodialysis. Several interconnected factors may explain the favorable outcomes observed in our pAVF cohort compared with historical surgical AVF benchmarks. First, hemodynamic considerations: pAVFs created with the Ellipsys system typically result in medium-flow fistulas with lower peak systolic velocities than surgically created brachial artery fistulas. Importantly, computational fluid dynamics studies have demonstrated that low and oscillating shear stress precisely localize the sites of intimal hyperplasia and subsequent stenosis in AVFs (18), suggesting that the more favorable flow profile in pAVFs may confer a biological advantage in reducing neointimal proliferation. Second, preservation of vascular anatomy: the percutaneous approach avoids extensive surgical dissection, thereby preserving the perivascular tissue and vasa vasorum, which may contribute to better vascular remodeling and long-term patency (19). Third, head-to-head clinical evidence: a recent long-term comparative study by Shahverdyan et al. (13) demonstrated that Ellipsys pAVFs achieved comparable or superior patency rates with fewer re-interventions compared with proximal forearm Gracz-type surgical AVFs, directly supporting the clinical relevance of these mechanistic advantages. Fourth, patient selection: as noted in the limitations, the pAVF cohort had a lower prevalence of prior AVF failure compared with patients who declined the procedure (23.4% vs. 50%), suggesting that favorable baseline vascular characteristics may have contributed to the observed outcomes. Fifth, procedural learning curve: our CUSUM analysis demonstrated that technical proficiency was achieved after approximately 30 cases (20), and outcomes improved progressively thereafter. This learning curve effect, rarely quantified in surgical AVF series, may partially explain why our results compare favorably with some historical surgical benchmarks.

The reviewer raises an important technical question regarding the minimum vein diameter threshold. In traditional open surgical AVF creation, a minimum vein diameter of 3 mm is generally recommended because the anastomosis is created by surgical incision and suturing, and the functional anastomotic area is directly proportional to the vein circumference (17). A smaller vein would result in an inadequate anastomotic area, leading to low flow and early failure. By contrast, the Ellipsys percutaneous system creates a side-to-side thermal fusion anastomosis between the artery and vein, with the anastomotic dimensions precisely controlled by the device (typically 2–3 mm in diameter). This geometry does not depend on the vein’s circumferential size in the same way as a surgical anastomosis. Furthermore, pAVFs are medium-flow fistulas (mean pump blood flow 312 ± 18 mL/min in our cohort), whereas surgical fistulas—particularly those involving the brachial artery—are typically high-flow conduits requiring larger venous inflow (18, 19). Finally, the flow-mediated dilation that occurs after AVF creation allows the 2.0–2.5 mm vein to enlarge progressively under increased shear stress. Seminal work by Malovrh demonstrated that even patients with small-caliber arteries (internal diameter ≤1.5 mm) achieved a nearly nine-fold increase in arterial blood flow within 3 weeks postoperatively, reaching adequate flow for dialysis by 8–12 weeks (21). This capacity for progressive vascular enlargement under flow stimulation explains why smaller veins can be acceptable for the percutaneous approach, provided the initial vessel quality is adequate (9, 15). These mechanistic differences explain why a lower vein diameter threshold is acceptable for the percutaneous approach.

In this study, the secondary intervention rate (29.7% of patients required intervention, average 0.41 per patient per year) was relatively low. This figure is lower than many reports on surgical arteriovenous fistulas (35–40% (7, 19)) but similar to other pAVF studies using similar devices (22, 23).

Our definition of functional maturation, which integrates successful repetitive cannulation and adequate dialysis delivery rather than mere Doppler flow velocity, provides a more clinically relevant benchmark for real-world practice. The median cannulation time of 42 days and the stable Kt/V values confirm that pAVFs not only meet radiological criteria but also translate effectively into reliable dialysis platforms. Regarding complications, we did not observe major complications such as steal syndrome, anastomotic pseudoaneurysm, or severe infection. This may be related to the anatomical location and creation principle of pAVF: the anastomosis is deep, and no artificial grafts are used, thereby reducing associated risks (15). The recorded complications were mainly cannulation-related issues and outflow stenosis, which are common problems with arteriovenous fistulas and can be effectively managed through routine monitoring and timely endovascular intervention. Notably, the absence of infection (0% incidence) is consistent with the device’s minimally invasive design and might offer advantages over surgical AVFs, which typically have a 2–5% infection rate (8).

The secondary intervention rate in our study (0.41 per patient-year) warrants comparison with established surgical AVF benchmarks. Shahverdyan et al. (15), in a head-to-head comparison of Ellipsys pAVF (*n* = 89) versus Gracz-type surgical AVF (*n* = 69), reported re-intervention rates of 0.86 versus 0.66 per patient-year, respectively. Our rate of 0.41 per patient-year is numerically lower than these reported benchmarks, which may reflect our center’s rigorous surveillance protocol and proactive endovascular management of developing stenoses before they progress to thrombosis. Importantly, these comparisons suggest that pAVF does not impose a higher intervention burden than traditional surgical AVF creation, and may offer comparable or even lower maintenance requirements in appropriately selected patients.

It should be noted that this study reflects the early learning curve of our center with this technique. As operator experience accumulates and understanding of the maturation process deepens (e.g., more aggressive early use of balloon angioplasty to manage anastomotic spasm or stenosis), technical success and maturation rates are expected to be further optimized (20). Based on our learning curve analysis, we suggest that each physician should complete at least 30 cases to master the key technical steps (e.g., vessel alignment, energy application) and achieve stable outcomes. This aligns with the ASDIN White Paper recommendation that proficiency in percutaneous AVF creation is typically attained after 25–35 procedures (20), providing a practical reference for centers adopting this technology.

This study has several inherent limitations that should be considered when interpreting our findings. First, the single-center, retrospective observational design lacks a concurrent control arm (e.g., traditional surgical AVF). Although we performed multivariate adjustments, residual confounding from unmeasured variables—such as surgeon experience in open surgery or detailed vascular calcification scores—cannot be entirely excluded. The absence of randomization precludes direct comparative effectiveness claims between pAVF and open surgery, and our findings should therefore be considered hypothesis-generating rather than confirmatory.

Second, selection bias is evident, as 10 patients who declined pAVF and underwent surgical creation were excluded; these patients had a higher prevalence of prior AVF failure (50% vs. 23.4%, *p* = 0.041), suggesting that the present cohort may represent a more favorable vascular access population, potentially overestimating the reported success rates. Furthermore, due to the retrospective nature of this study, the exact number of patients deemed anatomically ineligible (e.g., vessel diameters below the threshold or artery–vein distance >10 mm) could not be precisely ascertained from the available records, which further complicates the assessment of true non-candidacy rates and limits the generalizability of our results to unselected or complex vascular access patients.

Third, we acknowledge the risk of missing data and informative censoring. Although we achieved complete follow-up for the 64 included patients through June 2024, 6 patients were lost to follow-up and 4 had missing critical clinical parameters, necessitating their exclusion from the initial cohort. Furthermore, during follow-up, 5 patients died (cardiovascular events: *n* = 3; infection: *n* = 2) and 2 underwent successful kidney transplantation. These competing events could have overestimated patency rates if death or transplantation was associated with poor fistula function, though we have transparently reported the reasons for censoring in the results and found no significant baseline differences between censored and non-censored patients (all *p* > 0.05), suggesting that informative censoring was unlikely to substantially bias our estimates.

Fourth, although we applied the CIRSE classification system for complication grading and provided time-to-event estimates, the retrospective design and relatively small sample size limited our ability to perform formal competing risk analysis or to detect rare complications (e.g., steal syndrome) with sufficient statistical power.

Fifth, although we performed routine ultrasound surveillance, the relatively short follow-up duration (median 28 months) may not capture late complications such as aneurysm formation, which typically develop after 3–5 years of hemodialysis access use. Longer follow-up studies are needed to assess the true incidence of aneurysm and other long-term complications. Additionally, despite our rigorous chart review for Kt/V and cannulation records, minor adverse events (e.g., transient bruising, puncture-site pain) might be underreported due to the inherent limitations of retrospective data extraction. The intervention rate of 0.41 per patient-year, while compared with published surgical benchmarks, should be interpreted cautiously due to differences in study populations, surveillance protocols, and definitions of intervention across studies.

## 5 Conclusion

In summary, the practical experience from our center suggests that ultrasound-guided percutaneous arteriovenous fistula creation appears to be a safe, effective, and minimally invasive method for establishing hemodialysis access. It demonstrated excellent technical success, functional maturation, and patency rates during mid-term follow-up, with rare serious complications. Nevertheless, given the inherent limitations of our retrospective, single-center, non-comparative design, these findings should be considered hypothesis-generating rather than definitive. This technique may provide a valuable vascular access option for maintenance hemodialysis patients, particularly those with suitable vascular conditions or a preference for minimally invasive options. Future prospective multicenter studies with larger sample sizes, longer follow-up periods, and head-to-head comparisons are needed to further validate its long-term benefits and health economic value.

## Data Availability

The raw data supporting the conclusions of this article will be made available by the authors, without undue reservation.
